# Evaluation of Neighborhood Socioeconomic Characteristics and Advance Care Planning Among Older Adults

**DOI:** 10.1001/jamanetworkopen.2020.29063

**Published:** 2020-12-10

**Authors:** Sarah Nouri, Courtney R. Lyles, Anna D. Rubinsky, Kanan Patel, Riya Desai, Jessica Fields, Mindy C. DeRouen, Aiesha Volow, Kirsten Bibbins-Domingo, Rebecca L. Sudore

**Affiliations:** 1 Division of Palliative Medicine, Department of Medicine, University of California, San Francisco; 2 UCSF Center for Vulnerable Populations, Zuckerberg San Francisco General Hospital, San Francisco, California; 3 Department of Epidemiology and Biostatistics, University of California, San Francisco; 4 Division of Geriatrics, Department of Medicine, University of California, San Francisco; 5 Helen Diller Family Comprehensive Cancer Center, University of California, San Francisco; 6 San Francisco Veterans Affairs Medical Center, San Francisco, California

## Abstract

**Importance:**

Advance care planning (ACP) is low among older adults with socioeconomic disadvantage. There is a need for tailored community-based approaches to increase ACP, but community patterns of ACP are poorly understood.

**Objective:**

To examine the association between neighborhood socioeconomic status (nSES) and ACP and to identify communities with both low nSES and low rates of ACP.

**Design, Setting, and Participants:**

This cross-sectional study examined University of California San Francisco electronic health record (EHR) data and place-based data from 9 San Francisco Bay Area counties. Participants were primary care patients aged 65 years or older and living in the San Francisco Bay Area in July 2017. Statistical analysis was performed from May to June 2020.

**Exposures:**

Patients’ home addresses were geocoded and assigned to US Census tracts. The primary factor, nSES, an index combining area-level measures of income, education, poverty, employment, occupation, and housing or rent values, was divided into quintiles scaled to the distribution of all US Census tracts in the Bay Area (Q1 = lowest nSES). Covariates were from the EHR and included health care use (primary care, outpatient specialty, emergency department, and inpatient encounters in the prior year).

**Main Outcomes and Measures:**

ACP was defined as a scanned document (eg, advance directive), ACP *Current Procedural Terminology* code, or ACP note type in the EHR.

**Results:**

There were 13 104 patients included in the cohort—mean (SD) age was 75 (8) years, with 7622 female patients (58.2%), 897 patients (6.8%) identified as Black, 913 (7.0%) as Latinx, 3788 (28.9%) as Asian/Pacific Islander, and 748 (5.7%) as other minority race/ethnicity, and 2393 (18.3%) self-reported that they preferred to speak a non-English language. Of these, 3827 patients (29.2%) had documented ACP. The cohort was distributed across all 5 quintiles of nSES (Q1: 1426 patients [10.9%]; Q2: 1792 patients [13.7%]; Q3: 2408 patients [18.4%]; Q4: 3330 patients [25.4%]; Q5: 4148 patients [31.7%]). Compared with Q5 and after adjusting for health care use, all lower nSES quintiles showed a lower odds of ACP in a graded fashion (Q1: adjusted odds ratio [aOR] = 0.71 [95% CI, 0.61-0.84], Q2: aOR = 0.74 [95% CI, 0.64-0.86], Q3: aOR = 0.81 [95% CI, 0.71-0.93], Q4: aOR = 0.82 [95% CI, 0.72-0.93]. A bivariable map of ACP by nSES allowed identification of 5 neighborhoods with both low nSES and ACP.

**Conclusions and Relevance:**

In this study, lower nSES was associated with lower ACP documentation after adjusting for health care use. Using EHR and place-based data, communities of older adults with both low nSES and low ACP were identified. This is a first step in partnering with communities to develop targeted, community-based interventions to meaningfully increase ACP.

## Key Points

**Question:**

Do rates of advance care planning vary by neighborhood socioeconomic status (nSES)?

**Findings:**

In this cross-sectional study of 13 104 older adults in the San Francisco Bay Area, those living in neighborhoods with lower nSES had lower odds of advanced care planning compared with those in neighborhoods with high nSES, with those in the lowest quintile having 29% lower odds. Mapping geocoded data allowed identification of 5 San Francisco neighborhoods with both low nSES and advance care planning.

**Meaning:**

These findings may help to inform the development of multilevel interventions, including targeted, community-based program delivery to areas with the highest need and greatest disadvantage.

## Teaser

This cross-sectional study examines data from University of California San Francisco Health patients aged 65 years and older and whether there is an association between neighborhood socioeconomic status and rates of advance care planning.

## Introduction

Advance care planning (ACP), a process by which people communicate their preferences for future medical care,^1,2^ is associated with a higher likelihood of patients receiving care consistent with their goals and higher patient and family satisfaction with end of life care.^3,4,5,6^ However, ACP rates are as low as 20% to 30% among older, socioeconomically disadvantaged populations—including people of color and those with lower income—compared with rates higher than 50% among older adults overall. Rates of documented ACP in the electronic health record (EHR) are even lower.^7,8,9,10,11^ Known barriers to ACP include individual (eg, knowledge, attitudes) and health system level (eg, complexity of advance directive forms, lack of clinician training) factors. However, despite development of patient- and provider-facing programs to increase ACP,^12,13,14,15,16,17^ overall rates of ACP have not increased appreciably among diverse older adults in the US.^7^

ACP is a complex, socially mediated behavior^18,19^; thus, as with other socially mediated health behaviors, such as physical activity, blood pressure management, and tobacco use,^20,21,22^ it is likely that community-level factors influence ACP. More specifically, neighborhood socioeconomic status (nSES) has been associated with myriad health behaviors and social ties,^20,23^ among older adults in particular,^24,25,26,27,28^ and may therefore also be associated with ACP. Understanding community patterns of health behaviors, such as ACP, allows development of multilevel interventions, including targeted program delivery to areas with highest need and greatest disadvantage.^29^ Given persistently low rates of ACP among people of color and those with lower income,^10,11^ it is imperative to look beyond individual and health system level factors and engage communities in strategies to increase ACP.^30^

ACP is of critical importance now during the coronavirus disease 2019 (COVID-19) pandemic,^31,32^ particularly among communities with low nSES that not only are disproportionately affected by COVID-19’s health, social, and economic consequences, but also have higher morbidity and mortality in general.^33,34^ It is likely that these same communities with low nSES also have low ACP, but this remains unknown. Identifying communities with both low nSES and low ACP is the first step in developing community-based outreach, education, and intervention efforts to meaningfully increase access to ACP information and reduce disparities in ACP. In this study, we leveraged geocoded EHR data to examine the association between nSES and ACP and identify communities with both low nSES and low rates of ACP.

## Methods

### Study Population, Setting, and Geocoding

We included all University of California San Francisco (UCSF) Health patients who were empaneled, had a Bay Area address (defined as including the following 9 counties: Alameda, Contra Costa, Marin, Napa, San Francisco, San Mateo, Santa Clara, Solano, and Sonoma), and were aged at least 65 years as of July 7, 2017, which was the most recent date for which both empanelment and geocoded data were available. Per the UCSF Office of Population Health and Accountable Care (UCSF OPHAC), we defined empaneled patients as having a primary care practitioner (PCP) assigned in the electronic health record (EHR), and at least one in-person (noncanceled) or MyChart (patient portal) encounter in the prior 3 years. This study was approved by the institutional review board of UCSF, which waived informed patient consent because this study posed minimal risk to patients and could not be practically carried out without a waiver. This study follows the Strengthening the Reporting of Observational Studies in Epidemiology (STROBE) reporting guideline.

### Measures

Patients’ residential addresses as of July 7, 2017, were extracted from the EHR, geocoded to latitude and longitude coordinates using ArcGIS Business Analyst software version 2016 (Esri), then assigned to a 2010 US Census tract. Nearly all addresses (13 636 out of 13 677; 99.7%) were geocoded successfully. Patients with addresses outside the Bay Area or that we were unable to geocode were excluded from analysis.

We defined *ACP*, the outcome variable, as having at least one of the following documented in the EHR on July 7, 2017: (1) ACP scanned document (advance directive, living will, power of attorney, physician orders for life-sustaining treatment, do not resuscitate), (2) ACP *Current Procedural Terminology* codes (99497 or 99498), or (3) an ACP note type. We chose to include only EHR-documented ACP because documentation of ACP is essential for ensuring that the medical care provided to patients is consistent with their goals.^1,7^

We used a composite index^35^ of nSES at the census tract level as the primary factor variable. This index, previously developed through principal components analysis, incorporates the most recent 5-year averages from the 2013 to 2017 American Community Survey data on income, education, poverty, employment, occupation, housing and rent values, applied to 2010 US Census tract boundaries.^35^ Quintiles of nSES are based on the distribution of nSES across US Census tracts of SF Bay Area counties, with the first quintile (Q1) reflecting lowest nSES and the fifth quintile (Q5) reflecting highest nSES US Census tracts.

We extracted the following patient characteristics from the EHR: health care use (number of PCP, specialty outpatient, emergency department[ED], and inpatient encounters in the prior year), advanced illness (using a previously validated measure),^36^ cognitive impairment (defined by UCSF OPHAC using diagnostic and billing codes), and sociodemographic characteristics (age, sex, race/ethnicity, preferred language, insurance). Missing data were minimal: race/ethnicity data were missing for 288 patients (2.2%), and insurance data were missing for 36 patients (0.2%).

### Exploratory Mapping and Neighborhood Identification

Using ArcGIS software, we created a bivariable map of nSES and ACP, including only US Census tracts in San Francisco County with more than 5 patients. We chose to map only San Francisco County rather than all Bay Area counties because this is where the majority of patients in the study cohort lived, and therefore all US Census tracts in this county were represented. Because there are no thresholds for ACP that are meaningful from a clinical or quality perspective, we created ACP quintiles derived from our data to categorize US Census tracts by the percent of patients with ACP. US Census tracts were then assigned a value according to both their nSES and ACP quintiles, resulting in 25 unique values. We outlined US Census tracts on the map with the lowest 2 quintiles of ACP and nSES, allowing us to visualize potential clustering by neighborhood. We then identified neighborhoods (as defined by city and county government data)^37^ where more than 200 patients from the study cohort resided. We chose to describe the entire neighborhood rather than only the outlined US Census tracts because neighborhoods (rather than US Census tracts) would serve as the initial level of community engagement and partnership.

For these identified neighborhoods, and for San Francisco County overall as a reference, we used publicly available population survey data to describe sociodemographic characteristics including proportion of people aged 65 years or older, race/ethnicity categories, foreign-born, limited English proficiency, poverty among those aged 65 years or older, extremely low-income households, renter-occupied homes, and overcrowding. These data were obtained from the UCSF Health Atlas website, which synthesizes American Community Survey 2013 to 2017 estimates.^38^

### Statistical Analyses

We conducted descriptive analyses of the outcome variable, primary factor variable, and all patient characteristics. We used Pearson χ^2^ and Kruskal-Wallis tests to evaluate differences in ACP and patient demographic and health characteristics by nSES quintile. To assess the association between nSES and ACP while accounting for correlated data within US Census tract, we used mixed-effects logistic regression with a random intercept for US Census tract (unadjusted model). Because ACP is primarily introduced in clinical settings^18^ and our outcome variable in this study was documented ACP in the EHR (ie, requiring interaction with the health care system), we adjusted for health care use variables (adjusted model). Although we characterized nSES quintiles by patient characteristics in preliminary analyses, our evaluation of the association between nSES and ACP was limited to adjustment of health care use alone, because health care use accounts for much of the variation in clinical severity and appropriateness related to documented ACP. Because of historic and current institutional and systemic racism, nSES contributes in large part to racial/ethnic differences in health behaviors and outcomes. Therefore, we chose not to adjust for patients’ self-reported race/ethnicity or preferred language because racial/ethnic residential segregation has resulted in differential neighborhood-level advantage, social conditions, and community context.^39,40^ Furthermore, the association between neighborhood and individual-level factors is complex, and the latter may serve as mediators rather than confounders of neighborhood effects on health.^20,39^ We did not adjust for age because we restricted the population to include only those aged 65 years or older (a target population for ACP). We used a linear contrast test to assess a linear trend of documented ACP across nSES quintiles. For all analyses, a 2-sided *P* < .05 was defined as statistically significant. Stata software version 16 (StataCorp) was used to perform statistical analysis from May to June 2020.

## Results

### Patient Characteristics

The final study population included 13 104 patients after excluding 535 patients with addresses outside the Bay Area, 37 patients whose addresses could not be geocoded, and 1 patient who was assigned a pediatric primary care practitioner. Patient characteristics overall and by nSES quintile are summarized in Table 1. Of these patients, the mean (SD) age was 75 (8) years, 7622 (58.2%) were female patients, 897 (6.8%) identified as Black, 913 (7.0%) as Latinx, 3788 (28.9%) as Asian/Pacific Islander, and 748 (5.7%) as other minority race/ethnicity, and 2393 (18.3%) self-reported that they preferred to speak a non-English language. Within the prior year, 11 277 patients (86.1%) had had a primary care encounter, 11 072 patients (84.5%) had a specialty outpatient encounter, 1805 patients (13.8%) had an ED encounter, and 1340 patients (10.2%) an inpatient encounter. There were a higher proportion of White, English-speaking, and privately insured patients and a lower proportion of patients with ED and inpatient encounters in higher nSES quintiles (Table 1).

**Table 1.** ACP and Patient Characteristics by nSES Status Quintiles Scaled to the San Francisco Bay Area^a^

Characteristic	Patients, No. (%)					
	nSES quintiles, scaled to the Bay Area					Total
	1	2	3	4	5	
Total	1426 (100)	1792 (100)	2408 (100)	3330 (100)	4148 (100)	13 104 (100)
ACP	402 (28.2)	520 (29)	669 (27.8)	948 (28.5)	1288 (31.1)	3827 (29.2)
Any past-year encounter						
Primary care	1237 (86.7)	1564 (87.3)	2037 (84.6)	2871 (86.2)	3568 (86)	11 277 (86.1)
Specialty	1190 (83.5)	1529 (85.3)	1984 (82.4)	2812 (84.4)	3557 (85.8)	11 072 (84.5)
Emergency department	261 (18.3)	297 (16.6)	330 (13.7)	422 (12.7)	495 (11.9)	1805 (13.8)
Inpatient	181 (12.7)	223 (12.4)	224 (9.3)	336 (10.1)	376 (9.1)	1340 (10.2)
Age, mean (SD), y	76 (8)	75 (8)	75 (8)	75 (8)	74 (8)	75 (8)
Female patients	867 (60.8)	1122 (62.6)	1403 (58.3)	1944 (58.4)	2286 (55.1)	7622 (58.2)
Advanced illness	117 (8.2)	122 (6.8)	128 (5.3)	175 (5.3)	188 (4.5)	730 (5.6)
Cognitive impairment	439 (30.8)	496 (27.7)	547 (22.7)	774 (23.2)	978 (23.6)	3234 (24.7)
Race/ethnicity^b^						
White	350 (24.5)	613 (34.2)	1109 (46.1)	1692 (50.8)	2706 (65.2)	6470 (49.4)
Black	304 (21.3)	199 (11.1)	137 (5.7)	145 (4.4)	112 (2.7)	897 (6.8)
Latinx	130 (9.1)	219 (12.2)	188 (7.8)	196 (5.9)	180 (4.3)	913 (7)
Asian/Pacific Islander	532 (37.3)	629 (35.1)	795 (33)	1043 (31.3)	789 (19)	3788 (28.9)
Other	80 (5.6)	97 (5.4)	132 (5.5)	179 (5.4)	260 (6.3)	748 (5.7)
Missing	30 (2.1)	35 (2)	47 (2)	75 (2.3)	101 (2.4)	288 (2.2)
Preferred language						
English	966 (67.7)	1294 (72.2)	1913 (79.4)	2760 (82.9)	3778 (91.1)	10 711 (81.7)
Spanish	67 (4.7)	106 (5.9)	66 (2.7)	56 (1.7)	42 (1)	337 (2.6)
Chinese	240 (16.8)	165 (9.2)	238 (9.9)	305 (9.2)	175 (4.2)	1123 (8.6)
Korean	35 (2.5)	90 (5)	17 (0.7)	34 (1)	17 (0.4)	193 (1.5)
Vietnamese	26 (1.8)	37 (2.1)	53 (2.2)	44 (1.3)	25 (0.6)	185 (1.4)
Russian	22 (1.5)	25 (1.4)	36 (1.5)	27 (0.8)	22 (0.5)	132 (1)
Tagalog	16 (1.1)	31 (1.7)	23 (1.0)	18 (0.5)	17 (0.4)	105 (0.8)
Other	54 (3.8)	44 (2.5)	62 (2.6)	86 (2.6)	72 (1.7)	318 (2.4)
Insurance type^c^						
Private	289 (20.3)	491 (27.5)	767 (31.9)	1078 (32.5)	1302 (31.5)	3927 (30.1)
Medicare	1052 (73.8)	1230 (68.8)	1544 (64.3)	2137 (64.3)	2712 (65.6)	8675 (66.4)
Medi-Cal	84 (5.9)	67 (3.7)	91 (3.8)	106 (3.2)	118 (2.9)	466 (3.6)

Abbreviations: ACP, advance care planning; nSES, neighborhood socioeconomic status.

^a^ *P* < .001 for all except ACP (*P* = .03) and any past-year primary care encounter (*P* = .13).

^b^ Categories are mutually exclusive. Patients self-identifying as Hispanic or Latinx are included as Latinx.

^c^ Numbers do not sum to total and percentages do not sum to 100 due to 36 missing or other values. Private includes Medicare Advantage and Covered California.

The proportion of patients in each nSES quintile increased across higher quintiles, with 1426 patients (10.9%) in Q1 (lowest nSES quintile), 1792 patients (13.7%) in Q2, 2408 patients (18.4%) in Q3, 3330 patients (25.4%) in Q4, and 4148 patients (31.7%) in Q5. Only 3827 patients (29.2%) in the cohort had documented ACP: 28.2% (402 patients) in Q1, 29.0% (520 patients) in Q2, 27.8% (669 patients) in Q3, 28.5% (948 patients) in Q4, and 31.1% (1288 patients) in Q5. Patients lived in all 9 Bay Area Counties, but only in 968 of 1588 (61.0%) of US Census tracts in the Bay Area; the majority of patients (9094/13 104; 69.4%) lived in San Francisco County.

### Association Between ACP and nSES

In unadjusted analyses, patients living in US Census tracts with lower nSES (Q1-Q4) had lower odds of ACP documentation compared with those living in US Census tracts with the highest nSES (Q5), although this was only statistically significant for Q3 (odds ratio, 0.86; 95% CI, 0.75-0.98) and Q4 (odds ratio, 0.86; 95% CI, 0.76-0.98) (Table 2). After adjusting for health care use (ie, PCP, specialty outpatient, ED, and inpatient encounters in the prior year), lower quintiles of nSES were associated with progressively lower odds of ACP documentation (*P* for trend <.001). Specifically, compared with patients living in US Census tracts with the highest nSES, those in US Census tracts with lower nSES had significantly lower odds of ACP (Q1: aOR = 0.71 [95% CI, 0.61-0.84], Q2: aOR = 0.74 [95% CI, 0.64-0.86], Q3: aOR = 0.81 [95% CI, 0.71-0.93], Q4: aOR = 0.82 [95% CI, 0.72-0.93] compared with Q5).

**Table 2.** ACP by nSES in Unadjusted and Adjusted Analyses

nSES quintile	Patients, No.	ACP		
		Patients with documented ACP, No. (%)	Unadjusted analyses, OR (95% CI)	Adjusted analyses, aOR (95% CI)^a^
5	4148	1288 (31.1)	1 [Reference]	1 [Reference]
4	3330	948 (28.5)	0.86 (0.76-0.98)	0.82 (0.72-0.93)
3	2408	669 (27.8)	0.86 (0.75-0.98)	0.81 (0.71-0.93)
2	1792	520 (29.0)	0.87 (0.75-1.01)	0.74 (0.64-0.86)
1	1426	402 (28.2)	0.87 (0.75-1.02)	0.71 (0.61-0.84)

Abbreviations: ACP, advance care planning; aOR, adjusted odds ratio; nSES, neighborhood socioeconomic status; OR, odds ratio.

^a^ Model was adjusted for health care use (primary care, specialty outpatient, emergency department, and inpatient encounters in the prior year).

### Identification of Neighborhoods With Lowest nSES and ACP

US Census tracts representing the lowest 2 quintiles of nSES and ACP across San Francisco County are outlined in the Figure. Outlined US Census tracts clustered in 5 neighborhoods: Western Addition, the Mission, the Tenderloin, Excelsior, and the Bayview; 1501 (11.5%) empaneled UCSF patients aged 65 years and older live in these neighborhoods, and their rate of documented ACP ranges from 24.4% (70 of 287 patients) in the Tenderloin to 30.1% (96 of 311 patients) in the Mission (Table 3). On the basis of publicly available population survey data, 4 of 5 neighborhoods (Western Addition, Tenderloin, Excelsior, and Bayview) have a higher proportion of residents who are foreign-born compared with the San Francisco mean. Similarly, 4 of 5 neighborhoods (Western Addition, Mission, Tenderloin, and Bayview) have a higher prevalence of poverty among older adult residents and extremely low-income households compared with San Francisco averages. Notably, in the Tenderloin, 57% of households are extremely low-income (vs 22.3% in San Francisco) and 97.6% of homes are renter-occupied (vs 63.2% in San Francisco). Compared with San Francisco averages, Western Addition has a much higher proportion of older adults (20.6% vs 14.9%) and residents who identify as Black or African American (21.6% vs 6.4%). The Mission has a much higher proportion of residents who identify as Latinx (37.7% vs 15.3%) and are Spanish speaking (31.3% vs 11%). Excelsior has a higher proportion of residents identifying as Latinx (32.2% vs 15.3%) and Asian (51.4% vs 37.3%), and a much higher proportion with limited English proficiency (70.8%) than the San Francisco average (43.8%). Bayview has the highest proportion of residents identifying as Black or African American (30.3% vs 6.4%) among San Francisco neighborhoods.

**Figure.**
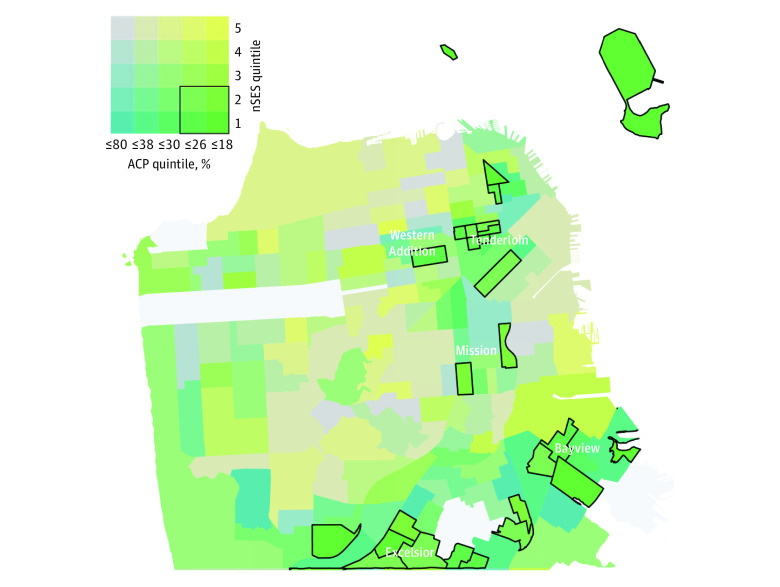
Bivariable Map of Advance Care Planning (ACP) and Neighborhood Socioceconomic Status (nSES) Quintiles in San Francisco County US Census Tracts With More Than 5 University of California San Francisco Primary Care Patients Aged 65 Years or Older ACP quintiles (1-5) were derived from our data and represent the percentage of patients in our cohort with ACP.

**Table 3.** Characteristics of Neighborhoods With Both Lowest Socioeconomic Status and Lowest ACP

Characteristics^a^	Patients, %					
	San Francisco county (n = 9094)^b^	Neighborhoods within San Francisco				
		Western Addition (n = 387)^b^	Mission (n = 311)^b^	Tenderloin (n = 287)^b^	Excelsior (n = 276)^b^	Bayview (n = 240)^b^
ACP, No. (%)^c^	2742 (30.2)	109 (28.2)	96 (30.1)	70 (24.4)	73 (26.5)	63 (26.3)
Age ≥65 y	14.9	20.6	10.3	14.5	15.5	12.0
Race/ethnicity^d^						
Non-Hispanic White	40.8	40.0	40.9	32.2	13.7	7.9
Black/African American	6.4	21.6	4.2	10.8	3.3	30.3
Latinx	15.3	8.8	37.7	24.6	32.2	23.2
Native American	1.9	2.3	1.5	2.0	1.3	0.8
Asian	37.3	30.9	17.1	31.0	51.4	38.1
Chinese	23.1	13.8	6.8	10.9	33.1	27.4
Filipino	5.5	3.0	3.8	6.3	14.1	3.7
Japanese	1.9	2.0	1.4	1.1	0.6	0.5
Korean	1.5	3.5	1.3	1.2	0.1	0.6
Vietnamese	2.2	3.5	1.2	4.9	2.4	4.6
Southeast Asian	8.7	7.2	5.6	14.1	18.3	9.6
Foreign-born	34.8	35.2	32.7	43.6	52.2	40.1
Limited English proficiency	43.8	39.0	43.8	50.6	70.8	56.0
Spanish speakers	11.0	3.8	31.3	18.9	26.1	19.4
East and Southeast Asian language speakers	25.7	22.2	7.8	22.1	41.9	34.3
Poverty among patients age ≥65 y^e^	13.6	19.1	19.0	35.8	10.3	16.1
Extremely low-income households^e^	22.3	35.0	23.3	57.0	19.2	34.4
Renter occupied	63.2	79.0	76.2	97.6	37.2	48.2
Overcrowding^f^	3.6	3.3	5.4	12.7	3.7	3.4

Abbreviation: ACP, advance care planning.

^a^ Data for neighborhood characteristics (excluding ACP) are obtained from the University of California San Francisco Health Atlas, which uses American Community Survey 2013 to 2017 estimates. American Community Survey estimates are weighted to bring sample characteristics in agreement with those of the full US population.

^b^ Number of empaneled University of California San Francisco patients aged 65 years or older living in San Francisco County or each neighborhood.

^c^ Number and percentage of empaneled University of California San Francisco patients aged 65 years or older in each neighborhood with documented ACP in the medical record.

^d^ Groups are not mutually exclusive.

^e^ Poverty is reported at 100% of the federal poverty level. Extremely low-income households are those that earn less than 30% of the area median income.

^f^ Overcrowding was considered to be households with more than 1.51 people per room.

## Discussion

This study found that compared with patients living in the highest SES neighborhoods, those in lower SES neighborhoods had lower odds of ACP; those in the lowest quintile had 29% lower odds. Using EHR and geocoded data, we identified socioeconomically disadvantaged communities of older patients with both the lowest nSES and the lowest rates of ACP. To our knowledge, this is the first study to use geocoded and nSES data to understand community patterns of ACP. This novel approach aims to understand where the most underserved older adults live (ie, in both neighborhood resources and ACP disparities) rather than using standard, individual-level sociodemographic characteristic data. This may allow for the development of tailored neighborhood-level ACP interventions.

Lower nSES was associated with lower odds of ACP after controlling for health care use. Currently, ACP is primarily introduced in clinical settings and to people with acute or progressive illness. Therefore, many community-dwelling older adults may be excluded, including those with less consistent access to medical care (eg, homebound, socially isolated, distrust of the health care system) and those who are healthier, despite a known preference among older adults for ACP to occur prior to acute or serious illness.^18,30,41,42^ Consistent with estimates of documented ACP among older adults in the US,^43,44,45,46^ we found similarly low rates of documented ACP. Notably, there was little absolute variation in ACP across nSES quantiles (28%-31%); this may be due to the substantial differences in health care use by nSES. For example, those living in US Census tracts with lower nSES were more likely to have inpatient or ED encounters where ACP is more likely to be documented than outpatient encounters. Given the low rates of documented ACP overall, our findings suggest that a focus only on a clinician or system-level model of ACP engagement may be inadequate in addressing disparities in ACP among underserved populations. ACP has been successfully promoted in faith-based community organizations for Black and Asian American populations, although these programs have not been widely implemented and may not reach entire communities.^47,48,49^ Our results underscore the importance of community-based strategies tailored to the needs of unique neighborhoods—to reach all community-dwelling older adults living in underresourced or disadvantaged communities.^29^

Given the complex etiology of health disparities and the sociocultural influences on end-of-life decision-making,^50^ it is important to consider community-based factors contributing to ACP disparities. The mechanism behind the association between nSES and ACP is likely multifactorial. Neighborhood SES has been associated with a wide range of health behaviors and outcomes among older adult populations with socioeconomic disadvantage .^20,24^ Disparities related to nSES may actually worsen for older adults over time because of the shrinking of their effective neighborhoods as they lose mobility with increasing age.^24,25,26^ nSES may also reflect the social (eg, sociocultural norms, distrust of the health system) and/or physical environment (eg, community-based organizations promoting ACP) distinct to a neighborhood.^24^ Alternatively, it is possible that the association between nSES and ACP is driven by individual-level SES; however, we could not ascertain individual-level SES from the EHR. Notably, the 5 neighborhoods highlighted in this study have a high proportion of residents of color and a high prevalence of poverty, in large part due to residential redlining (ie, the systematic denial of federal, local, or private resources by area), resulting in racial segregation that has prevented wealth accumulation among people of color.^51,52^ These same neighborhoods have higher rates of preventable hospitalizations, are more susceptible to effects of climate change,^53^ and are at higher risk of gentrification.^54^ All of these factors have been shown to influence socially mediated health behaviors, such as ACP.^40,51,54,55^ Although our analyses cannot differentiate between these possible mechanisms, they nevertheless reveal that community patterns of ACP differ by nSES.

Leveraging diverse data sources to identify neighborhoods with lowest SES and ACP is an essential first step in developing tailored solutions at the neighborhood level. Community-based strategies have proven successful for other health efforts, including HIV and cancer prevention,^56^ and may hold similar promise for ACP.^30^ Identifying these neighborhoods will help identify community partners and advisors with which to conduct formative research to explore community-specific ACP needs^57^ and to develop and implement community-based interventions. In light of the COVID-19 pandemic, it has become increasingly apparent that low SES and predominantly Black and Latinx communities have faced both higher mortality and greater social and economic distress.^33,34,58^ This underscores the tantamount importance and urgency of ensuring person-centered, high quality ACP and end-of-life care.

### Limitations

This study has several limitations. The data are from a single health system in the Bay Area, with a high proportion of patients in high nSES US Census tracts, and many US Census tracts in the Bay Area not represented in our cohort. Our approach suggests we can identify low ACP by neighborhood characteristics and determine neighborhoods to target for community-based interventions; however, replication in other health systems and communities is needed. Rates of ACP were low among older adults across all quintiles of nSES. It is possible that a greater proportion engaged in ACP that was not documented in the UCSF EMR or was documented in another health system.^59^ However, we purposefully limited our population to patients who were empaneled, and therefore presumably engaged with our health system.

## Conclusions

This study’s findings suggest that low nSES is associated with low ACP documentation after adjusting for health care use. Using EHR and place-based data, we identified socioeconomically disadvantaged communities of older adults with both low nSES and low ACP. Doing so will allow our team and others to identify neighborhood partners and advisors to develop targeted, community-based interventions to increase ACP engagement. Next steps include developing and testing neighborhood-based ACP interventions and examining ACP rates across communities longitudinally. Future research can attempt to incorporate data across local health systems and regions to better reflect the local population. Given the association between nSES and ACP, and the disproportionate health disparities of diverse older adults for morbidity and mortality, future research to better understand and delineate community-level effects on ACP is essential.
